# Effectiveness of scoliosis-specific exercises for alleviating adolescent idiopathic scoliosis: a systematic review

**DOI:** 10.1186/s12891-020-03517-6

**Published:** 2020-07-27

**Authors:** Yunli Fan, Qing Ren, Michael Kai Tsun To, Jason Pui Yin Cheung

**Affiliations:** 1 Department of Orthopaedics and Traumatology, The University of Hong Kong – Shenzhen Hospital, 1 Haiyuan 1st Road, Futian District, Shenzhen, Guangdong China; 2 Department of Physiotherapy, The University of Hong Kong-Shenzhen Hospital, Shenzhen, Guangdong province China; 3 Department of Orthopaedics and Traumatology, The University of Hong Kong, 5/F Professional Block, Queen Mary Hospital, Pokfulam, Hong Kong SAR, China

**Keywords:** Adolescent idiopathic scoliosis, Scoliosis specific exercise, Cobb angle, Truncal asymmetry, Quality of life

## Abstract

**Background:**

Adolescent idiopathic scoliosis (AIS) is the most common pediatric spinal deformity with reported complications including pain, mental health concern and respiratory dysfunction. The scoliosis-specific exercise (SSE) is prescribed throughout pubertal growth to slow progression although effects are unclear. This review aims to establish the effectiveness of SSE for alleviating AIS in terms of reducing Cobb angle, improving trunk asymmetry and quality of life (QoL). Additionally, it aims to define the effects of age, skeletal maturity, curve magnitude and exercise compliance on the outcomes of SSE.

**Methods:**

A systematic reviewed was conducted to net SSE articles. Searched databases included PubMed, MEDLINE, Cochrane Library, Scopus, CINAHL and Google scholar. The quality of study was critically appraised according to the PEDro scale.

**Results:**

A total of ten trials with an average PEDro score of 6.9/10 were examined in this study. Two randomized controlled trials (RCTs) and two clinical controlled trials suggested that SSE alone and with bracing or traditional exercise had clinical significance in reducing Cobb angle more than 5°. One RCT specifically implicated no comparable effects between bracing and SSE in prevention of curve progression for moderate scoliosis. There was insufficient evidence to support the positive effects of SSE on improving truck asymmetry (*n* = 4) and QoL (*n* = 3). Five studies evaluated the interaction effects of age (*n* = 2), skeletal maturity (*n* = 1) and curve magnitude (n = 2) with SSE in reducing Cobb angle yet without drawing any firm conclusions.

**Conclusions:**

Insufficient evidence is available to prove that SSE with or without other conservative treatments can reduce Cobb angle, improve trunk balance and QoL. The interaction effects of age, skeletal maturity, curve magnitude, and exercise compliance with SSE in reducing Cobb angle are not proven. Future studies should investigate the relationship of influencing factors and SSE in treating AIS but not only testing its effectiveness.

**Trial registration:**

INPLASY202050100.

## Background

Adolescent idiopathic scoliosis (AIS), characterized by lateral deviation, axial rotation, and abnormal sagittal curvature of the spine, is the most common (70–80%) spinal deformity with unclear etiology [1]. Its prevalence is approximately 0.47–5.2% in the general adolescent population [2]. This condition may lead to cosmetic concerns [3], pain [4], and respiratory dysfunction [5]. AIS was reported in almost 10% of patients requiring either conservative or surgical treatment [6]. Surgery is reserved for severe curves of 50°, whereas bracing and scoliosis-specific exercise (SSE) are reserved for mild (10°–25°) and moderate (25°–45°) curves to prevent progression to the operative stage [7].

Bracing is the most common conservative treatment if the Cobb angle is > 25° in patients with growth potential [8–10]. It produces an external pushing force to straighten the trunk and to derotate the rib cage. Skeletal maturity [8], in-brace correction [11], curve magnitude [12], flexibility [13–15] and compliance with brace wearing [16] are significant factors influencing the outcomes of brace treatment. However, bracing can be stressful for patients, induce a flatter back [17], and negatively affect quality of life (QoL) [18–23]. Additionally, most braces are uncomfortable to wear, resulting in poor brace-wearing compliance [24]. In contrast, SSE is commonly accepted by patients [25]. Moreover, SSE is recommended alone or as an add-on to bracing for preventing scoliosis progression [26]. Several techniques of SSE have been established in previous studies [27–32]; some techniques are described more often than others [33]. Although the method used varies, all techniques adhere to the same principle, namely: 1) three-dimensional self-correction; 2) training activities of daily living; and 3) stabilization of corrected postures [34]. Updated studies have reported promising effects of SSE on curve regression [35–48], which warrants a thorough investigation.

The latest review concluded that no valid evidence proved the effect of SSE on curve progression prevention [49]. In particular, three studies have used the same cohort [43–45]; one was a single-arm prospective study without a comparative untreated group [42]. Regarding the National Health and Medical Research Council (NHMRC) hierarchy of evidence [50], a randomized control trial (RCT) is considered the best methodology for answering intervention questions in a literature review. Thus, the quality of enrolled studies in that review was relatively poor [49]. Another three systematic reviews enrolled studies between 2005 and 2017 and found insufficient and low-quality clinical trials showing effects of SSE on improving the scoliotic deformity [33, 51, 52]. One review confirmed the promising effects of the Schroth method in curve regression but had analyzed only four studies [51]; one review analyzed nine articles of which three (33%) did not use SSE and one (11%) was an outdated article published 15 years ago [33]. One study analyzed eight articles of which three (37.5%) were rated as being low quality (PEDro score: 3), one (12.5%) had a retrospective study design, and 50% (*n* = 4) were published 10 years ago [52]. A number of controlled trials have been published after 2017, which calls for an updated systematic review.

Based on currently available evidence, SSE may be effective for improving spinal deformity; however, this is supported by only low-quality evidence. Moreover, unlike bracing, no review discussed the influencing factor of SSE on scoliotic curvature improvement. Understanding how SSE functions is crucial rather than accepting its effectiveness. Therefore, this review aims to access the most updated SSE studies that adhered to the Society on Scoliosis Orthopedic and Rehabilitation Treatment (SOSORT) exercise principle [34] to evaluate the effect of SSE on scoliotic deformity improvement. Moreover, we aim to define the effects of age, skeletal maturity, curve magnitude, and exercise compliance on SSE outcomes.

## Methods

### Search strategy

**Fig. 1:**
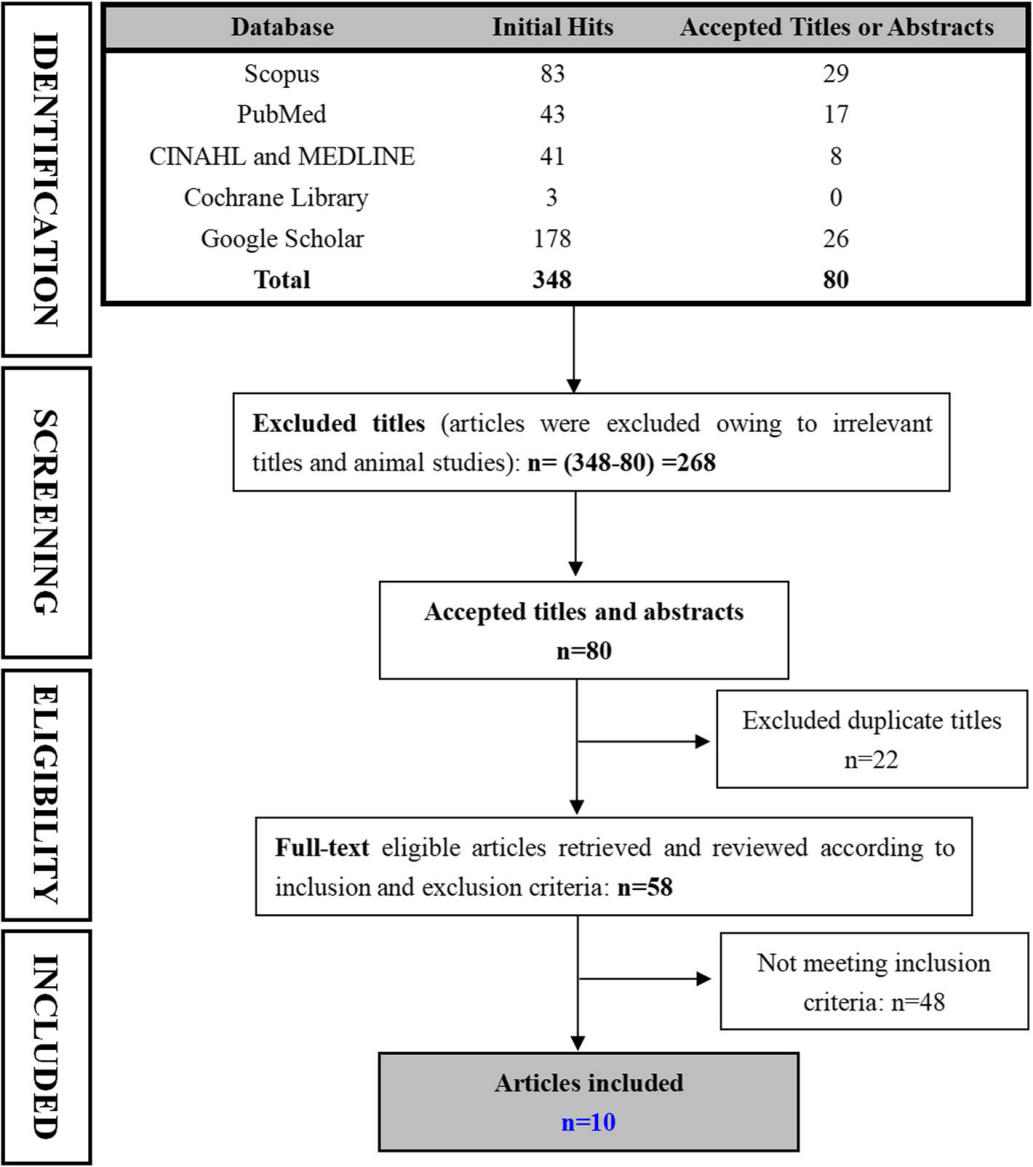
Search flow chart

This systematic review replicated the search strategy adopted by the Cochrane Review from January 1, 2010 to February 29, 2020 in the following six databases: PubMed, MEDLINE, Cochrane Library, Scopus, CINAHL, and Google Scholar (Fig. 1). Key search items consisted of “AIS”, or “idiopathic scoliosis”, and “exercise”, or “scoliosis specific exercise”, or “physiotherapy”, or “Schroth”, or “SEAS”, or “DoboMed, or “Side-shift” or “FITs” or “randomi*” or “placebo” or “control*”. These included subject headings, text words, methodological terms, disorder terms, and treatment terms, and all are listed in full in the search strategy in Additional file 1. This review protocol was registered on the INPLASY.COM with registration number INPLASY202050100. Searched results from each database were cross-checked by two independent researchers. Potentially relevant abstracts were screened based on the inclusion criteria, and full-text articles were obtained for eligible results. The two researchers discussed any disagreements regarding accepting full-text articles until consensus was achieved.

### Inclusion criteria

The PICOS principle was applied to set the inclusion criteria, specifically described as: 1) P (population): adolescents with idiopathic scoliosis, 2) I (intervention): reported any of the SSE methods in either study or control group, 3) C (comparison): compared with traditional exercise, no treatment, standard care, brace, or any other non-SSE, 4) O (outcome): Cobb angle was reported in degrees as the primary outcome to evaluate effects on curve regression, with or without the secondary outcome defined as the truncal asymmetry (angle of trunk rotation in degrees: ATR) or condition-related function/QoL measured using validated questionnaires (e.g. 22-item or 23-item Scoliosis Research Society questionnaire), and 5) S (study design): prospective studies with controls that were published in or after 2010 were included.

### Exclusion criteria

Any animal/pharmacological study, retrospective human clinical trial, or prospective single-arm study written in a language other than English or published before 2010 was excluded.

### Evidence hierarchy and methodological appraisal

The NHMRC hierarchy of evidence was adopted to evaluate the evidence level [50]. Level II evidence (RCT) was considered the best methodology to answer intervention-related questions in a systematic review. However, considering the limited number of RCT in the most up-to-date reviews [33, 49, 51, 52], prospective clinical control trials (CCT: Level III) were also analyzed in this study.

Methodological qualities were measured using the PEDro scale [53]. The PEDro scale was proven to have validity and reliability for evaluating the methodological quality of clinical trials [53]. It has been commonly used to evaluate physiotherapy studies [54]. The PEDro scale scores methodology based on 10 items: 1) random allocation, 2) concealed allocation, 3) similarity at baseline, 4) subject blinding, 5) therapist blinding, 6) assessor blinding, 7) > 85% follow-up for at least one outcome, 8) intention-to-treat analysis, 9) between-group comparison for at least one outcome, and 10) point and variability measures for at least one outcome (Table 1). Items were scored as either present [1] or absent (0). A score out of 10 ranked the study as having weak (PEDro score: 0–4), moderate (PEDro score: 5–7), and strong (PEDro score: 8–10) quality.

**Table 1:**
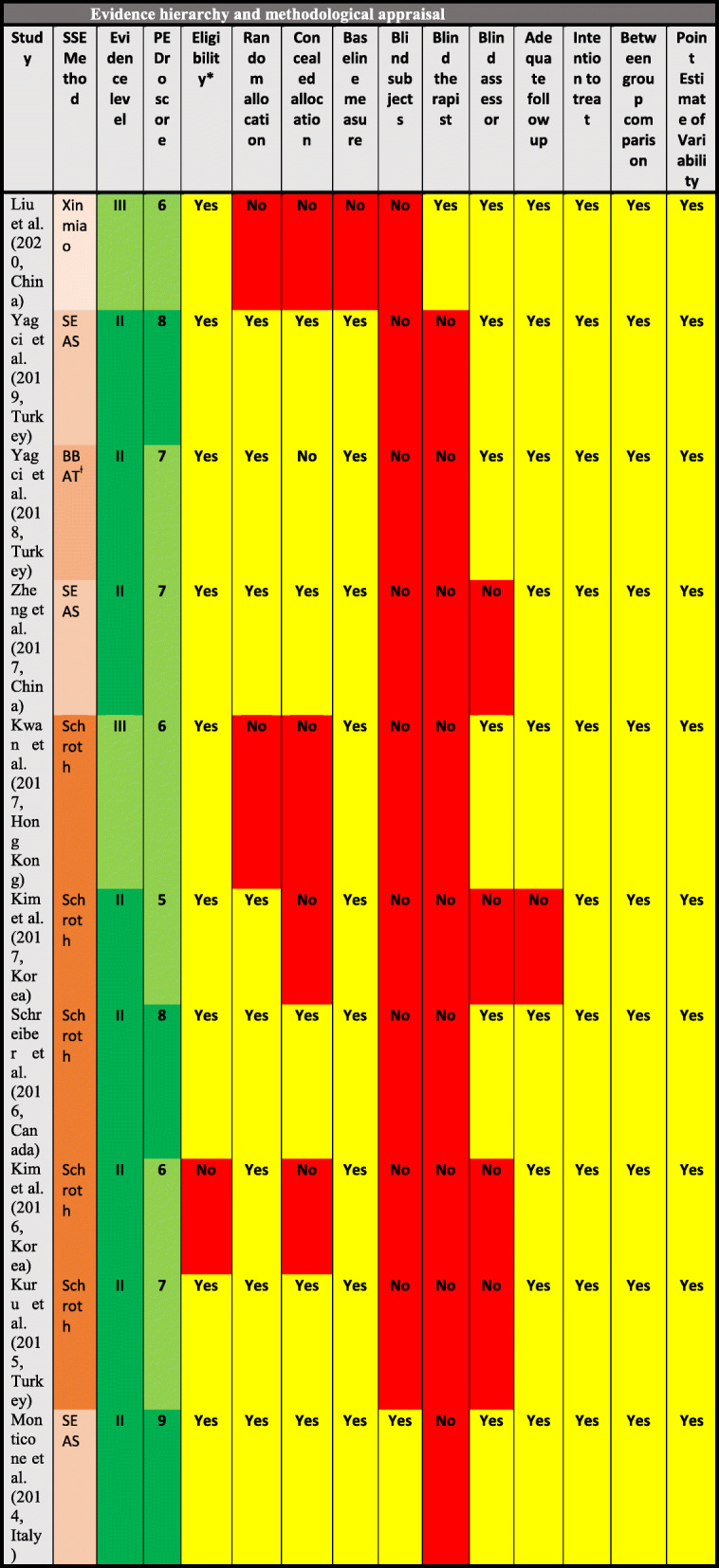
Evidence hierarchy and methodological appraisal

## Results

### Search results

A total of 348 initial hits were obtained from six databases. In total, 268 unrelated and 22 duplicate titles were excluded after their titles and abstracts were screened (Fig. 1). Up to 48 of 58 full-text articles were excluded because of the following reasons: 1) ineligible study designs (single-arm study: *n* = 8, retrospective study: *n* = 5, observational study: *n* = 2, and feasible study: *n* = 3); 2) incorrect interventions (head positioning: *n* = 1, general stretching: n = 5, core muscle training: *n* = 7, spinal manual therapy: n = 3, electrostimulation therapy: *n* = 2, and traction: n = 1); 3) inappropriate patient population (n = 3, adult participants); 4) inappropriate outcomes (n = 2, Cobb angle was not compared); 5) full-text was not in English (*n* = 4); and 6) duplicates (n = 2, same cohort with multiple publications). Finally, 10 articles were included in this review (Fig. 1).

### Evidence hierarchy and methodological appraisal

Eight articles with an RCT methodology (80%) were classified as providing level II evidence [35–37, 41, 45–48], and two articles with prospective CCT methodology (20%) were classified as providing level III evidence [38, 40] (Table 1). The PEDro scale was ranked from 5 to 9, with an average score of 6.9/10 for overall articles (Table 1). Specifically, the average scores of Schroth studies [35–38, 45], scientific exercise approach to scoliosis (SEAS) studies [41, 47, 48], and alternative SSE [40, 46] studies were 6.4 (*n* = 5, score: 5–8), 8 (*n* = 3, score: 8–9), and 6.5 (*n* = 2, score: 6–7), respectively. Criteria 4 (blinding subjects) and 5 (blinding therapists) were not met for 80% (*n* = 8) of studies [35–38, 45–48]. However, one RCT [41] reported participant blinding, whereas one CCT [40] reported therapist blinding.

### Characteristics of included studies

Five trials adopted the Schroth method (Table 2). In particular, three RCTs compared the Schroth method alone with standard care [45], Pilates [35], and home exercise [37]; one CCT compared the Schroth method and bracing with bracing alone for moderate scoliosis [38]; one RCT compared the Schroth method and respiratory exercises with the Schroth method alone for mild to moderate scoliosis [36]. Three trials adopted the SEAS method (Table 2). These included one RCT comparing SEAS with core stabilization exercises in patients with moderate scoliosis [47], one RCT comparing SEAS with bracing for moderate scoliosis [48], and one comparing SEAS with traditional exercises for mild scoliosis [41]. Another two studies adopted alternative SSE (body awareness, and Xinmiao approach) [40, 46]: one was an RCT comparing traditional exercises and body awareness exercises with traditional exercise alone for both mild and moderate scoliosis [46]; a CCT study grouped participants by age (< 10 years, 10–12 years, and 13–15 years) to determine the relationship of age, skeletal maturity, and gender with intervention effects [40].

**Table 2:** Study characteristics

Study	Study Design	Sample Size	Gender		Age (mean ± SD)/ range	Initial Cobb angles	Curve magnitudes	Bone Maturity (Risser sign)	Intervention Protocols	
			Female	Male					Study group	Control group
**Liu et al** [40]	CCT	99	66	33	7–15	10°-24°	3 Rt. thoracic, 10 Lt. thoracic, 4 Rt. Lumbar, 13 Lt. Lumbar, 49 Rt. Thoracic with Lt. Lumbar, 20 Lt. Thoracic with Rt. lumbar	0: *n* = 37 1–2: *n* = 23 3: *n* = 39	**Alternative SSE method (Xinmiao):** corrective postures + corrective exercise: 40 min/session, education for 2 days then independently performed daily at home.	**No control**
**Yagci et al** [46, 47]	RCT	30	30	0	12–16	20°-45°	Rt. thoracic Lt. Lumbar: *n* = 16 Single thoracolumbar: *n* = 14	2: n = 14 3: *n* = 16	Brace + SEAS* Supervised session: 40 min/session; once per week Home Program: 20 min/day	**Brace + core muscle strengthen exercise** same dosage with the study group
**Yagci et al** [46, 47]	RCT	20	20	0	10–16	20°-45°	King classification: type 1: n = 5, type 2: *n* = 10, type 3: n = 3, type 4: n = 2	1: n = 2 2: *n* = 11 3: n = 7	**Basic Body Awareness Therapy (BBAT) + Traditional Exercise + brace:** home exercise 1 h/session, 5sessions/week	**Traditional Exercise only + brace** same dosage with the study group
**Zheng et al** [48]	RCT	53	41	12	10–14	21°-36°	Not reported	Not Reported	**SEAS:** supervised exercise in clinic: 40 min/week + home exercise: 10-15 min/day	**Brace:** 23 h/day
**Kwan et al** [38]	CCT	48	38	10	10–14	25°-40°	Thoracic major: 21% vs 33% Thoracolumbar/lumbar major: 79% vs 67%	0–1: 54% vs 79% 2: 29% vs 17% 3–5: 17% vs 4%	**Brace + Schroth:** 8-week outpatient program + home exercise + revisit every two months + 18 h/day of bracing.	**Brace alone:** 18 h/day
**Kim et al** [6, 35]	RCT	15	10	5	13–23	16°-40°	Not reported	Not reported	**Schroth + Respiratory muscle exercise:** 15 min of Resp + 40 min Schroth/session; 3 sessions/week;	**Schroth only:** 1 h/session; 3 sessions/week
**Schreiber et al** [43, 44, 45]	RCT	50	47	3	13–14	10°-45°	3C: n = 7 3CP: *n* = 15 4C: n = 5 4CP: n = 23	Mean: 1.76 vs 1.44	**Schroth:** 5 × 60 min of outpatient sessions delivered first 2 weeks, then 60 min 1×/week + 30 to 45 min of daily home sessions for 6 months.	**Standard care**, consisting of observation or bracing if the SRS bracing criteria were met.
**Kim et al** [6, 35]	RCT	24	24	0	14–17	10°-27°	Not reported	Not reported	**Schroth:** 60 min, 3x/week for 12 weeks.	**Pilates exercise:** 60 min, 3x/week for 12 weeks.
**Kuru et al** [37]	RCT	45	39	6	11–14	20°-50°	Not reported	Mean: 1.5 vs 1.4 vs 1.0	**Group 1: supervised Schroth + asymmetric position rotational breathing:** 1.5 h/day, 60 min, 3x/week for 6 weeks + home program. **Group 2: home Schroth exercise + asymmetric position rotational breathing,** 18 sessions for 6 weeks.	**Observation only.**
**Monticone et al**	RCT	110	80	20	10–14	10°-25°	Thoracic: n = 16 Lumbar: *n* = 27 Thoracolumbar: *n* = 41 S-shaped: 26	0: *n* = 50 1: *n* = 60	**SEAS+ cognitive behavioral strategies + ergonomic education.** 60 min of outpatient sessions delivered once a week + 30 min of home sessions 2x/week. Mean time on treatment = 42.8 months (SD 9.1).	**General exercises for spinal mobilization.** 60 min of outpatient sessions. Once a week + 30 min of home program sessions 2x/week at home.

3C: a thoracic curvature without pelvis imbalance. 3CP: a thoracic curvature with pelvis imbalanced. 4C: a thoracolumbar/lumbar curvature without pelvis imbalance. 4CP: a thoracolumbar/lumbar curvature with pelvis imbalanced

Variations in intervention dosage were found, from daily to every other day (Table 2). Four trials reported > 1-year follow-up, whereas six trials had study periods of 2–6 months (Table 3). However, only five studies reported exercise compliance in percentage values of prescribed dosage (Table 3).

**Table 3:** Result summary

Study	Study period	Exercise compliance	Outcome measurements				Results	Evaluation of interaction effects			
			Cobb	Trunk asymmetry	QoL	Others		Age	Bone maturity	Curve magnitudes	Exercise compliance
**Liu et al.**^**a,**^**b**[40]	2.08 years	Not Reported	√	×	×	×	1.Significant Cobb reduction (6.8 ± 5.5° vs 1.5 ± 4.8°, *p* < 0.01) between the group A (age < 10 years, Risser 0) and the group C (age > 13 years, Risser 3). 2. Significant Cobb reduction was noted in subjects aged 10–12 (3.1 ± 4.2°).	Yes (* < 13 years)	Yes (*Risser: 0)	Unknown	N/A
**Yagci et al.** [46, 47]	4 months	Study: 64% Control:62%	√	√ ATR POTSI WRVS	√ SRS-22 (initial mean: 4.0)	×	1. D-value of Cobb: −5.3 ± 2.2° vs −4.8 ± 2.6° in thoracic; − 4.1 ± 2.5° vs − 3.5 ± 3.0 in lumbar; *P* > 0.05 between groups. 1. No difference of ATR, POTSI, WRVAS between groups. 2. Pain domain was only improved in the control group (4.7 vs 4.3).	Unknown	Unknown	**Yes** **No difference**	Unknown
**Yagci et al.**^**a,b**^ [46, 47]	10 weeks	Study:69% Control:88%	√	√ ATR POTSI WRVS	√ SRS-22 (initial mean:3.9)	×	1. Significant difference of D-value in Thoracic Cobb reduction between groups (− 7.33 ± 2.78° vs 0.63 ± 4.34°). 2. Body asymmetry was only improved in the study group (ATR: −4.3 vs − 4.2). 3. SRS-22 was unchanged in both groups.	Unknown	Unknown	Yes (* Thoracic)	Unknown
**Zheng et al.**^**a**^ [48]	1 year	Study: 59 ± 0.2% Control: 58 ± 0.27%	√	√ Shoulder-balance TAPS ATI	√ SRS-22 (initial mean:4.2)	×	1. Greater Cobb reduction in the bracing group (5.58 ± 6.37° vs 2.24 ± 3.19°). 2. Shoulder balance was only improved in the bracing group. 3. Significant difference of functional domain (4.9 vs 4.7), mental domain (4.5 vs 4.2) between groups.	Unknown	Unknown	N/A	Unknown
**Kwan et al.**^**a,b**^ [38]	18.1 ± 6.2 months	Study:77% Control:79%	√	√ ATR	√ SRS-22 (initial mean:4.2)	×	1. 17% improved, 62% stabilized and 21% worsened in the study group; 4% improved, 46% stabilized and 50% worsened in the control group. 2. No difference in the ATR between groups. 3. The SRS-22 result favored the exercise group (function domain: 4.8 vs 4.6).	Unknown	Unknown	Unknown	Unknown
**Kim et al.**^**a**^ [6, 35]	8 weeks	Not reported	√	×	×	√ Pulmonary function	1. Significant Cobb reduction between groups (D-value of Cobb angle: 4.26 ± 1.36 vs 2.69 ± 1.11, p < 0.05) 2. Significant peak expiratory flow between groups (D-value of PEF: −1.30 ± 0.87 vs −0.17 ± 0.68, *p* < 0.05)	Unknown	N/A	N/A	N/A
**Schreiber et al.**^**a**^ [43, 44, 45]	6 months	Supervised:76%. Home program: 73%	√	×	×	×	1. Significantly (D-value of Cobb: −3.5° vs + 2.3°, p < 0.01) smaller largest curve and sum of curves (decreased 0.4°, p < 0.05) between groups.	Unknown	Unknown	Unknown	Unknown
**Kim et al.**^**a,b**^ [6, 35]	3 months	Not reported	√	×	×	√ Weight distribution	1. Significant (P < 0.05) inter−/intra-group Cobb reduction: Schroth group: 23.6 ± 1.5° to 12.0 ± 4.7°; Pilate group: 24.0 ± 2.6° to 16.0 ± 6.9° 2. No difference of weight distribution in the control whereas significant (*P* < 0.05) improvement was noted in the study group.	Unknown	Unknown	N/A	N/A
**Kuru et al.**^**a**^ [37]	24 weeks	Not reported	√	√ ATR, height of hump, waist-asymmetry	√ SRS-23 (initial mean:3.9)	×	1. Greater improvements in Cobb angle (−2.53°, *P* < 0.001), ATR (−4.23°, P < 0.001), height of hump (−68.66 cm, P < 0.01) and waist asymmetry (P < 0.01) were observed in the supervised exercise group compared with the home exercise group and control at 24-week. 2. No difference of QoL between groups (4.4 vs 3.9 vs 4.1).	Unknown	Unknown	N/A	N/A
**Monticone et al.**^**a,b**^	42.8 months	Not reported	√	√ ATR	√ SRS-22 (initial mean:3.8)	×	1. Significant (−5.3° vs + 1.7°, p < 0.001) improvement of Cobb angles between groups at skeletal maturity and at 1-year follow up. 2. Significant difference of ATR between groups (−3.5° vs −0.4°, *p* < 0.001). 3. The Seas group showed significant (P < 0.001) improvements in all domains of the SRS-22 at skeletal maturity (1-year follow-up: 4.8 vs 4.0).	Yes (* ≥ **13 years)**	Unknown	Unknown	N/A

^a^ Significant intra−/inter-group Cobb reduction. ^**b**^ Significant Cobb reduction beyond 5°in the SSE group. *QoL* Quality of Life *POTSI* Posterior Trunk Symmetry Index, *WRVS* Walter Reed Visual Assessment Scale, *TAPS* Trunk Appearance Perception Scale, *ATR* angle of trunk rotation, *ATI* angle of trunk inclination. √: reported in the either methodology or results. × Not reported. *Yes*, the interaction effect with SSE was discussed. Unknown: not discussed, *N/A* not applicable

Six studies compared the truncal asymmetry pre- and post-intervention (Table 3). Four of them showed that SSE was not superior to core exercises, traditional exercises, and bracing for ATR improvement [38, 46, 47] or shoulder balance [48]. Two studies showed better improvement of ATR in the study group [37, 41]. For QoL (Table 3), five studies adopted the SRS-22 questionnaire [38, 41, 46–48], and one study adopted the SRS-23 questionnaire [37]. However, a high initial score (mean score: 3.8–4.2) was noted in all studies (Table 3), with three studies reporting better QoL outcomes in terms of function and mental domain, favoring the SSE group [41, 46, 48]. Two studies found no significant differences of QoL between the groups in either adding SSE to bracing treatment or comparing supervised SSE with home exercises [37, 46]. Another study found improved pain domain outcomes in the core exercise group only [47].

### Proposed questions

1. Can SSE improve scoliotic deformity?

Ten studies with 494 participants were enrolled in this review (Table 2). Five trials (three RCTs and two CCTs) with moderate study quality showed significant curve regression in terms of reducing Cobb angles beyond the measurement error of 5° (Table 3). Three studies [35, 40, 41] enrolled participants with mild scoliosis (Cobb angle: 10°–27°): Monticone et al. reported a decrease of 5.3° with SEAS but an increase of 1.7° with general exercise at skeletal maturity [41]; Kim et al. found a large curve regression from 23.6 ± 1.5° down to 12.0 ± 4.7° in the Schroth group, whereas a reduction from 24.0 ± 2.6° to 16.0 ± 6.9° was observed in the Pilates group after 3 months of exercises [35]; Liu et al. grouped participants according to age and revealed that notable curve regressions were favored in patients younger than 13 years (a decrease of Cobb angle: 6.8 ± 5.5° for age < 10 years; 3.1 ± 4.2° for age 10–12 years) and with Risser stage 0 (a decrease of Cobb angle: 5.7 ± 5.6°) at 2-year follow-up [40]. Another two studies involved brace-wearing patients with moderate scoliosis [38, 46]. Kwan et al. found that 17% of the participants showed improvement with Schroth exercises, whereas only 4% improved with no exercise at 1.5-year follow-up [38]. Yagci et al. adopted body awareness exercise with bracing and revealed a significant Cobb angle reduction (− 7.33 ± 2.78° vs 0.63 ± 4.34°, *p* < 0.05) of the thoracic curvature between the groups [46].

Three RCTs showed statistically significant reductions in Cobb angle, but differences were not of clinical significance (Table 3). Kim et al. conducted an 8-week-long study and reported a reduction of Cobb angle by 4.26 ± 1.36° after Schroth with respiratory exercises [36]. Schreiber et al. conducted a 6-month-long study and demonstrated a 3.5° decrease in the largest curves but only a decrease of 0.4° in the sum of curves (root mean square value) with Schroth therapy [45]. Kuru et al. performed a 6-month-long study with three groups and found greater Cobb angle reduction (− 2.53°, *p* = 0.03) in those who relied on supervised Schroth therapy [37].

Furthermore, two RCTs concluded that SSE was not superior than either bracing or core muscle exercises in improving scoliotic deformity (Table 3). Yagci et al. compared the SEAS with core muscle exercises in participants wearing a brace and revealed comparable effects between the thoracic (− 5.3 ± 2.2° vs 4.8 ± 2.6°) and lumbar (− 4.1 ± 2.5° vs − 3.5 ± 3.0°) curvatures [47]. Zheng et al. compared the SEAS alone with bracing for moderate scoliosis and suggested that a notable reduction in Cobb angle only favored the bracing group (bracing: 5.58 ± 6.37° vs SEAS: 2.24 ± 3.19°, *p* = 0.01) [48].

2. Effects of age, skeletal maturity, curve magnitude, and exercise compliance with SSE in reducing Cobb angle.

Two studies investigated the relationship between age and intervention effects [40, 41]. One RCT with high study quality (PEDro: 9) revealed that in the SEAS group, participants aged ≥13 years had better results than younger patients (− 5.8° vs − 4.8°) [41]. One CCT with moderate study quality (PEDro: 6) revealed the opposite result in terms of better outcomes (− 6.8 ± 5.5°/− 3.1 ± 4.2° vs − 1.5 ± 4.8°), favoring younger patients (< 13 years) [40].

One study analyzed the interaction effect of skeletal maturity, in the form of Risser sign, with SSE in improving scoliotic deformity [40]. This study suggested that subjects with Risser stage 0 significantly benefited from SSE in curve regression compared with those with Risser stage 3 (Risser stage 0: 5.7 ± 5.6° vs Risser stage 3: 2.1 ± 4.7°).

Two studies compared decreasing values in Cobb angle between thoracic and lumbar curves (Table 3). One study demonstrated that only body awareness therapy could significantly improve thoracic curvatures, and yet, no difference was detected in comparison with the traditional exercise group [46]. Another study revealed that both thoracic and lumbar Cobb angles decreased in all participants wearing braces regardless of the exercise strategy (SEAS vs Core muscle training) [47].

No study investigated the correlation of exercise compliance with intervention effects. Five studies reported exercise compliance in the percentage value of the prescribed dosage (Table 3: 58 ± 0.27 to 88%) [38, 45–48], of which three trials reported significant intergroup differences in Cobb angles that were beyond measurement error [38, 46, 48]: one study showed greater Cobb angle reduction in patients undergoing brace with exercise [38]; another study showed that bracing was superior to exercise alone for moderate scoliosis [48]; and the third study suggested body awareness exercise with bracing could effectively improve scoliosis [46].

## Discussion

This review aimed to estimate the effect of SSE on scoliotic deformity improvement. Unlike the previous reviews [33, 51, 52], besides reporting a reduction in Cobb angle, our review emphasized the true effect in terms of reductions beyond clinical measurement errors. The clinical standard for individual curve regression was reported to be > 5° [55]. Therefore, any change within or equal to 5° was not considered as a true improvement. The most updated meta-analysis revealed that few RCTs can be used for effect size estimation [33], of which only three SSE studies (two with a low risk of bias [41, 45] and one with a high risk of bias [35]) showed a mean reduction of only 5° (D-value of Cobb: − 8.95°, − 1.05°); three studies showed greater reductions but with a high risk of bias [56–58]. Moreover, these three studies adopted no typical SSE (core muscle training [56], posture education [57], and traditional exercise therapy [58]) to compare with untreated control therapies. Hence, this meta-analysis concluded that only low-quality evidence is available to suggest that SSE improves spinal deformity [33]. Regarding the lack of studies to perform meta-analysis, our study comprehensively reviewed the most recent trials to estimate the effectiveness and clinical importance of SSE in reducing Cobb angle.

According to our review, five studies with moderate to high quality (PEDro: 6–9) reported a significant decrease in Cobb angle beyond 5° (Table 3). Three studies involved patients with moderate scoliosis, and thus, bracing was included as an intervention strategy. Specifically, two studies adopted underarm orthosis [38, 46], and one study did not report brace type [48]. Nonetheless, two of them consistently suggested that bracing with SSE was superior to bracing alone or with traditional exercise to treat moderate scoliosis [38, 46]. Another study implied that SSE could not replace bracing to treat moderate scoliosis due to the lack of comparable effects between the two methods [48]. However, none of the studies reported the initial in-brace correction. Therefore, the results can be challenged if the baseline in-brace correction is not evenly distributed. Additionally, a study demonstrated that SSE reduced correction loss during the bracing period [59], which indicated that the role of SSE during bracing was maintaining in-brace correction. However, without reporting the initial in-brace correction, it is not possible to determine whether SSE enhanced or maintained in-brace correction. Therefore, the role of SSE during the bracing period requires further study. Two studies considered as moderate to high quality (PEDro: 6–9) in this review compared SSE alone with traditional care for mild scoliosis [40, 41]. They consistently suggested that SSE had significant effects on a curve regression for mild scoliosis until skeletal maturity. This is promising as curves < 30° are unlikely to progress after skeletal maturity [5]. Therefore, the findings of these two studies are of clinical value and encouraging for patients with mild scoliosis to commence SSE. Consequently, due to the limited number of eligible studies available, insufficient evidence is available to prove the effect of SSE on curve regression for mild scoliosis.

For secondary outcomes, five trials compared truncal asymmetry pre- and post-exercise, of which three studies consistently found that SSE was not superior to brace or other exercises in improving either ATR or shoulder balance for brace-wearing patients [38, 47, 48]. SSE was only effective for improving truncal asymmetry in patients with moderate scoliosis if used as a supplement to body awareness exercise [46]. Improvement of ATR was noted if applied to patients with only mild scoliosis [41]. In particular, two studies adopted the Posterior Trunk Symmetry Index and the Walter Reed Visual Assessment Scale [46, 47]. One study used the angle of trunk inclination and trunk appearance perception with quantifying shoulder balance [48], and two studies used ATR only [38, 41] to quantify changes in trunk asymmetry. Therefore, insufficient evidence is available to support the effects of SSE on truncal asymmetry improvement. In addition, a standardized algorithm is ideal for evaluating trunk asymmetry.

Six studies evaluated QoL pre- and post-interventions (Table 3). This review revealed a high initial score in all studies, which is consistent with a previous meta-analysis [51]. Thus, investigating the effects of SSE on each domain is valuable for providing a clear understanding of where the effects lie, which can assist physiotherapists in determining which strategy should be implemented to achieve specific goals. Four trials in this review studied one domain each [38, 46–48]. Two studies were conducted by Yagci et al.; they showed that the pain domain only improved with core muscle training exercise during bracing [46, 47]. Two studies [38, 48] similarly revealed that better QoL outcomes relied on either functional or mental domains (Table 3). Hence, insufficient evidence is available to append the benefits of SSE in improving QoL.

The influencing factors for brace treatment were in-brace correction, skeletal maturity, curve magnitude, and brace compliance [60]. However, this remains undefined for SSE treatment. Therefore, this is the first review to estimate the interactions between SSE and these factors. This is clinically valuable for physiotherapists to set individualized exercise protocols and estimate prognosis for patients undertaking SSE. However, only four studies addressed this concern. One study in particular revealed that better Cobb angle reduction was achieved in patients aged ≥13 years [41], whereas a recent study revealed the opposite result [40]. This inconsistency may be explained by different exercise approaches and varied acceptance of SSE in different countries. Additionally, it was intriguing that 34.3% (*n* = 34) of the patients with atypical AIS (10 left thoracic, 4 right lumbar, and 20 left thoracic with right lumbar) were recruited in that recent study [40]. Therefore, the result should be interpreted with caution when demonstrating the effects of SSE on the AIS population. One study investigated the relationship of skeletal maturity and intervention effects and suggested that better outcomes occur in patients with early Risser stages [40]. One study found that body awareness exercise with bracing is better at correcting thoracic curves. However, this study ended prematurely at the tenth week, which is a limitation because the curve can deteriorate again after a short follow-up [26]. Up to 50% of the studies in this review reported exercise compliance with a percentage value of the prescribed dosage. Although all mentioned articles consistently state that exercise adherence is crucial, no information was available to evaluate the interactions between compliance and SSE outcomes. This knowledge gap must be addressed in the future.

The main limitation of the review is the lack of high-quality studies, which makes it difficult to extract adequate data to reach any firm conclusions. The previous systematic reviews [33, 52], analyzed the same five studies [35, 37, 41, 45, 48] included in our review, revealed a significant heterogeneity by statistical testing and concluded that no pooled effect sizes could be reliably reported. In addition, our review included five more updated clinical trials that showed notable methodological heterogeneity: one CCT was conducted with a large sample size (*n* = 99) but no comparative untreated controls [40]; four studies, including one CCT [38] and three RCTs [36, 46, 47], were conducted with small sample sizes (*n* < 50) which could mask variations and build up of systematic errors. Moreover, those four studies [36, 38, 46, 47] in which all included bracing strategy, introduced confounding effects of bracing and SSE in treating AIS. Therefore, considering the notable heterogeneity of enrolled studies, a meta-analysis was not performed in this review. However, this review suggested that SSE has a significant effect on Cobb angle reduction, which concurred with previous reviews [34, 52]. Additionally, this review has implications for researchers identifying knowledge gaps in this field. More RCTs are required to clarify the role of SSE as a treatment for moderate AIS during bracing. In addition, the best SSE type for different curve types as well as the most effective protocol (frequency and intensity) among those available should be determined. Moreover, the key factors that influence the success of exercise treatment should be elucidated. To achieve this goal, multicenter studies with matched groups of participants are required in the future.

## Conclusions

Limited evidence with moderate quality suggested that SSE can significantly reduce Cobb angle and improve truck asymmetry. The effect of SSE with brace wearing on treating moderate scoliosis is unclear. Insufficient evidence is available to implicate any effects of SSE on changes in QoL. This is the first review to study the factors influencing the success of SSE treatment, which remains undefined and requires further investigation.

## Supplementary information

**Additional file 1 Appendix 1:** Search strategy.

## Data Availability

Data sharing is not applicable to this article as no datasets were generated or analysed during the current study.

## Abbreviations

AIS: Adolescent idiopathic scoliosis
SSE: Scoliosis-specific scoliosis
QoL: Quality of life
SRS: Scoliosis research society
SEAS: Scientific exercise approach to scoliosis
POTSI: Posterior Trunk Symmetry Index
WRVS: Walter Reed Visual Assessment Scale
TAPS: Trunk Appearance Perception Scale
ATR: Angle of trunk rotation
ATI: Angle of trunk inclination
