# Supplementary material for: Effectiveness of scoliosis-specific exercises for alleviating adolescent idiopathic scoliosis: a systematic review
Source: BMC Musculoskelet Disord. 2020 Jul 27;21:495. doi: 10.1186/s12891-020-03517-6 (PMC7385878; doi:10.1186/s12891-020-03517-6)
Supplement: Supplementary file 1 — Additional file 1 Appendix 1: Search strategy. [file 12891_2020_3517_MOESM1_ESM.docx]

**Appendix 1:** Search strategy

1. PubMed
2. Physical Therapy Modalities[mh] 2. Physiothera* [tw] 3. Conservative Treatment[mh] 4. Conservat*[tw] 5. Complementary Therapies[mh] 6. Complementary*[tw] 7. complement Pathway, Alternative[mh] 8. Alternat*[tw] 9. Specific[tw] 10. Medicine, Traditional[mh] 11. Traditional[tw] 12. Exercise[mh] 13. Exercise*[tw] 14. Schroth*[tw] 15. SEAS*[tw] 16. Side-shift*[tw] 17. FIT*[tw] 18. Lyon* [tw] 19. Or/1-18 20. Scoliosis[mh] 21. 22. *Scoliosis[tw] 23. 20 OR 21. 24. 19 AND 22. 25. animal[mh]. 26. humans[mh] 27. NOT/25-26.

2. MEDLINE

1. randomized controlled trial.pt. 2. controlled clinical trial.pt. 3. randomized.ab. 4 placebo.ab,ti. 5. drug therapy.fs. 6. randomly.ab,ti. 7. trial.ab,ti. 8. groups.ab,ti. 9. or/1-8 10. (animals not (humans and animals)).sh. 11. 9 not 10. 12. Comparative Study/ 13. exp Randomization Studies/ 14. exp Controlled Studies/ 15. exp Prospective Studies/ 16. exp Cross-Over Studies/ 17. exp Epidemiologic Studies/ 18. exp Case-Control Studies/ 19. exp Cohort Studies/ 20. exp Cross-Sectional Studies 21. (cohort adj (study or studies)).mp. 22. cohort analy.mp. 23. (follow up adj (study or studies)).mp. 24. exp Spinal Diseases/ 25. exp Scoliosis/ 26. Idiopathic scoliosis.mp. 27. or/24-27 28. exp Exercise 29. Scoliosis-specific exercis.mp. 30. exp Exercise Therapy/ 31. exp Schoth/ 32. exp SEAS/ 33. FITs.mp. 34. exp Side-shift/ 35. or/28-34 36. 26 and 29 and 4

3. Cochrane Library

1. MeSH descriptor Spinal Diseases explode all trees. 2. MeSH descriptor Scoliosis explode all trees. 3. Scoliosis. 4. (1 OR 2 OR 3). 5. MeSH descriptor Exercise explode all trees. 6. exercis*. 7. MeSH descriptor Exercise Therapy explode all trees. 8. MeSH descriptor Scoliosis Specific Exercise explode all trees. 9. MeSH descriptor Schorth explode all trees. 10. physiotherap*. 11. MeSH descriptor SEAS explode all trees. 12. MeSH descriptor Side-shift explode all trees. 13. MeSH descriptor Side-shif explode all trees. 14. MeSH descriptor Dobomed explode all trees. 15. MeSH descriptor FITs explode all trees. 16. (OR/5-15). 16. (4 AND 16)

4. Scopus

S7: S6 and S1. S6: S5 or S4 or S3 or S2. S5: DE "Exercise" (Explode thesaurus term). S4: DE "PHYSICAL therapy" (Explode thesaurus term). S3: DE "Scoliosis-specific exercise" (Explode thesaurus term). S2: conservati*. S1: scoliosis.

5. CINAHL

S45: S32 and S37 and S44. S44: S38 or S39 or S40 or S41 or S42 or S43. S43: (MH "Conservat+.S42: "physiotherapy”. S41: (MH "Physical Therapy+"). S40 (MH "Therapeutic Exercise+"). S39: exercise*. S38 (MH "Exercise+"). S37: S33 or S34 or S35 or S36. S36 "scoliosis". S35: (MH "Scoliosis"). S34: (MH "Spinal Diseases+"). S33: (MH "Spine+"). S32: S30 NOT S31. S31: (MH "Animals"). S30: S26 or S29. S29: S27 or S28. S28: observational N1 stud*. S27: cohort N1 stud*. S26: S7 or S12 or S19 or S25. S25: S20 or S21 or S22 or S23 or S24. S24: volunteer*. S23: Prospecti*. S22: control*. S21: follow-up stud*. S20: follow-up stud*. S19: S13 or S14 or S15 or S16 or S17 or S18: S18 (MH "Prospective Studies+"). S17: (MH "Evaluation Research+"). S16: (MH "Comparative Studies"). S15: latin square. S14.(MH "Study Design+"). S13: (MH "Random Sample"). S12: S8 or S9 or S10 or S11. S11: random*. S10 placebo*. S9: (MH "Placebos"). S8: (MH "Placebo Effect"). S7: S1 or S2 or S3 or S4 or S5 or S6. S6: triple-blind. S5: single-blind. S4: double-blind. S3: clinical W3 trial. S2: "randomi?ed controlled trial*". S1: (MH "Clinical Trials+").

6. Google Scholar

Simple search in titles and abstracts for "scoliosis" and “exercise”.
